# Introduction *Highlights of the Asthma Summit 2009: Beyond the Guidelines*

**DOI:** 10.1097/WOX.0b013e3181d0ff01

**Published:** 2010-02-15

**Authors:** Michael A Kaliner, Judith R Farrar

**Affiliations:** 1 Institute for Asthma and Allergy, Wheaton and Chevy Chase, MD, and George Washington University School of Medicine, Washington, DC; 2 LifeSciences Press, Canandaigua, NY

## 

Asthma affects more than 22 million persons in the United States, including more than 6 million children. In the past 2 decades, the gap between science and clinical practice has been narrowed by better understanding of asthma pathophysiology and improvements in therapeutic approaches, which have been well described in national and international management guidelines.[1,2] Nonetheless, the disease remains a substantial burden for patients, families, and health-care systems.

Current guidelines call for treatment decisions to be based on assessments of disease severity.[1,2] These assessments must be regular, as asthma is not a stable or static disease. Furthermore, assessment questions must be specific and address the 2 components of disease severity. One is impairment, which is characterized by symptoms, nighttime awakenings, changes in normal activities, lung function, and use of short-acting *β*-agonists for symptom control. The second is risk, defined as the propensity for exacerbations, treatment-related side effects, and progressive loss of lung function over a period of time. Although impairment can be quantified effectively and is well addressed by current treatment approaches, the same cannot be said for risk. However, risk will have a more profound effect on patients' disease over time.

There are differences among the major asthma guidelines, and whether the differences can be (or need to be) reconciled is not yet clear. If so, the next question is whether guidelines should be universal and standardized internationally.

These questions were the focus of interesting, and sometimes controversial, dialogue at the inaugural Asthma Summit held in Baltimore, MD, in February, 2009. International key opinion leaders, physicians, and scientists convened to discuss state-of-the-art issues in asthma genetics, pharmacotherapeutics, and clinical management. Entitled, Beyond the Guidelines, the Summit explored how to "operationalize" the concepts of asthma control, dosing flexibility, and heterogeneity of disease to help clinicians improve patient care.

Chaired by William Busse, MD, a distinguished faculty of international prestige, including Jean Bousquet, MD; Chris Brightling, MD; William Calhoun, MD; G. Walter Canonica, MD; Gene Colice, MD; Frederick Hargreave, MD; Erwin Gelfand, MD; and Stephen Lazarus, MD, presented the most current clinical aspects of their research. The research presentations were followed by interactive panels, workshops, and debates with the goal of applying the findings to everyday patient issues. The program was developed by a steering committee that included Bradley Chipps, MD; Peter Dicpinigaitis, MD; Michael Kaliner, MD; Allan Luskin, MD; Sheldon Spector, MD; and William Storms, MD.

It is evident that many patients with asthma want to reduce their medications, but whether treatment can be flexible has been a matter of debate. In addition, new treatment approaches, such as those that address early life events, multiphase aspects of inflammation, or allergic factors, have not been explored fully. Consideration of these questions and further improvement of asthma management will require a view of asthma not as a single disease, but as one with many ramifications and end points. Furthermore, although guidelines provide direction on how to treat patients with asthma, it is likely that treatments will have to become more patient specific. These are some of the issues discussed by faculty and attendees at the Asthma Summit, which are captured in the following articles and discussion.

## Notes

These Proceedings are sponsored by LifeSciences Press, LLC, and supported by an unrestricted educational grant from Sepracor, Inc.

All content has been derived from the Asthma Summit 2009, which was sponsored by SRxA Institute for Professional Education and Medical Education Resources and funded by unrestricted educational grants from Sepracor, Inc.; Abbot Laboratories; Graceway Pharmaceuticals; Strategic BioSciences; and the TREAT Foundation.
